# Evaluation of *Candida albicans* and anaerobic bacterial co-expansion in vulvovaginal candidiasis: a cross-sectional study

**DOI:** 10.3389/fmicb.2026.1939688

**Published:** 2026-09-03

**Authors:** Luigi Tordelli Ruda, Loris Bertoldi, Natalia Pedretti, Luca Spaggiari, Samyr Kenno, Samuele Sabbatini, Elena Roselletti, Silvia Bozza, Roberta Gaziano, Eva Pericolini, Andrea Ardizzoni, Claudia Monari

**Affiliations:** 1 Department of Medicine and Surgery, University of Perugia, Perugia, Italy; 2 BMR Genomics s.r.l., Padova, Italy; 3 HIP-TECH PhD Program, University of Modena and Reggio Emilia, Modena, Italy; 4 Department of Surgical, Medical, Dental and Morphological Sciences with Interest in Transplant, Oncological and Regenerative Medicine, University of Modena and Reggio Emilia, Modena, Italy; 5 Department of Experimental Medicine, University of Rome “Tor Vergata”, Rome, Italy

**Keywords:** anaerobic bacteria, *C. albicans*, CST, vaginal microbiota, VVC

## Abstract

**Background:**

Vulvovaginal candidiasis (VVC) is a common mucosal infection predominantly attributed to *Candida albicans* (*C. albicans*). However, this yeast frequently colonizes the vaginal niche without causing symptoms, indicating that additional microbial and host factors might contribute to disease onset and/or pathology. This study investigated whether VVC is associated with a shift in the vaginal microbiota.

**Methods:**

Vaginal samples from 96 non-pregnant women were stratified into three study groups: healthy (NEG, *n* = 54), asymptomatic (ASYMP, *n* = 20), and symptomatic (SYMP, *n* = 22). Vaginal pH, PMN infiltration, and *C. albicans* morphology in vaginal samples were assessed microscopically, while fungal load was quantified by qPCR and isolates were tested for hyphal formation and biofilm production. Vaginal microbiota was analyzed by 16S rRNA amplicon sequencing on a subset of 43 representative vaginal samples.

**Results:**

Most SYMP women exhibited a characteristic inflammatory profile, with 75% showing a vaginal pH ≥ 4.5, 95.45% presenting marked PMN infiltration (score 2) and all displaying filamentous *C. albicans* forms *in vivo*. In contrast, 90.91% of ASYMP women had a vaginal pH 4.5, none showed PMN infiltration (score 0), and 65% displayed the fungus exclusively in the yeast form. The vaginal burden of *C. albicans* DNA was 4.9-fold higher in SYMP than in ASYMP women. Of interest, symptomatic disease was associated with the selective co-enrichment of the anaerobic bacteria *Dialister micraerophilus* and *Aerococcus christensenii*, which emerged as the main microbial features distinguishing SYMP from ASYMP women. Notably, the relative abundance of *Dialister micraerophilus* positively correlated with both PMN infiltration score and vaginal pH.

**Conclusion:**

These findings identify *Dialister micraerophilus* and *Aerococcus christensenii* as key anaerobic bacterial signatures associated with VVC. Further studies are needed to assess the possible role of these bacteria in VVC causation or modification.

## Introduction

Fungal infections represent a significant public health concern, characterized by rising incidence and substantial morbidity, particularly when misdiagnosed or inadequately treated (Bongomin et al., 2017; Denning, 2024). Fungal infections affecting the oral cavity, gastrointestinal tract, and female genital tract are the most common clinical manifestations. Vulvovaginal candidiasis (VVC), mainly caused by *Candida albicans* (*C. albicans*) is the most widespread fungal infection globally, with an estimated 70–75% of reproductive-age women experiencing at least one episode during their lifetime (Sobel et al., 1998); and 5–8% develop recurrent VVC (RVVC). RVVC affects nearly 138 million women annually, underscoring the limitations of current antifungal strategies (Denning et al., 2018).

While *C. albicans* is the primary agent of VVC, it is also a commensal component of the healthy vaginal microbiota, with asymptomatic colonization rates of up to 33% in premenopausal individuals (Sobel, 2007; Achkar and Fries, 2010). In this commensal state, *C. albicans* exists predominantly as yeast and does not elicit a host immune response. However, specific environmental and host-related conditions, such as antibiotic misuse, immunosuppression, diabetes (O’Laughlin and McCoy, 2023), hormonal fluctuations, or disruption of the vaginal microbiota, can trigger the transition to a pathogenic hyphal form (Achkar and Fries, 2010). This morphological switch is central to VVC pathogenesis, as hyphae exhibit increased adhesive and invasive properties, releasing aspartyl proteases (i.e., SAP4-6) and candidalysin, which induce a robust inflammatory response responsible for the clinical symptoms (Gaziano et al., 2023; Rautemaa-Richardson et al., 2026).

The human vaginal environment relies on complex and dynamic interactions between microbial communities and the host. In healthy, reproductive-aged women, the microbiota is typically dominated by *Lactobacillus* spp., including *L. crispatus*, *L. jensenii*, *L. gasseri* and *L. iners* (Ma et al., 2012). The composition of the vaginal microbiota has been categorized into five Community State Types (CSTs) (Ravel et al., 2011), where CST I (*L. crispatus*-dominated) is strongly associated with vaginal health (Verstraelen et al., 2009; Alonzo Martínez et al., 2021) due to low pH maintenance, immune modulation and production of antimicrobials that inhibit *C. albicans* (Verstraelen et al., 2009; Breshears et al., 2015; Parolin et al., 2015, 2022; Niu et al., 2017; Reid, 2018; McKloud et al., 2021). Conversely the role of CST III (*L. iners-*dominated) in promoting vaginal health or dysbiosis is still debated (Verstraelen et al., 2009; Breshears et al., 2015; Alimena et al., 2022). Recent data from an Italian cohort revealed a high abundance of *L. iners* in healthy individuals, suggesting geographical variations within Europe, highlighting the need to account for population context (Vinerbi et al., 2025). A significant reduction in *Lactobacillus* spp. is associated with adverse outcomes, including bacterial vaginosis, preterm birth, infertility, and increased susceptibility to sexually transmitted infections (Ling et al., 2010; Nasioudis et al., 2017; Ceccarani et al., 2019; Han et al., 2021), leading to the hypothesis that dysbiosis predisposes women to VVC. However, the role of *Lactobacillus* spp. in VVC onset remains inconclusive and, in some cases, controversial. Some studies report that women with VVC maintain a *Lactobacillus*-dominant microbiota comparable to healthy controls (Zhou et al., 2009; Macklaim et al., 2015), while others report a significant decrease in *Lactobacillus* spp. abundance during VVC (Ceccarani et al., 2019; Liang et al., 2024; Zhang et al., 2024). This reduction has been linked to an increase in facultative anaerobes like *Gardnerella vaginalis* and *Prevotella* spp. Furthermore, *L. crispatus* is frequently replaced by *L. iners* during VVC, and our group previously demonstrated that *L. iners* cell-free supernatants enhance *C. albicans* biofilm formation, and hyphal and pseudohyphal development *in vitro* (Sabbatini et al., 2021).

This study aims to investigate vaginal microbiome alterations in women with VVC compared to *C. albicans*-colonized asymptomatic carriers and *C. albicans*-negative healthy controls, identifying specific bacteria potentially associated with VVC.

## Methods

### Study design, ethics statement, and patient cohort

This cross-sectional, observational study was conducted in accordance with the Declaration of Helsinki and approved by the Ethical Committee of Umbria, Italy (MICROCA-CEAS ref. 3070/17; CA2020-CER ref. 3802/19). All participants provided written informed consent. A sample of 167 women aged 18–53 years referred to the Microbiological Diagnostic Service of the University Hospital “Santa Maria della Misericordia,” Perugia, Italy, was recruited between: February–October 2018 and December 2021–October 2022.

Subject eligibility was based on a standardized clinical questionnaire. Vulvovaginal candidiasis (VVC) diagnosis required specific vulvovaginal signs and symptoms paired with *Candida* spp. isolation. Main exclusion criteria included active menstruation and postmenopausal state.

To ensure the evaluation of a cohort specifically affected by *Candida*-associated vulvovaginitis, subjects with vulvovaginitis caused by (or concomitant with) other pathogens, including *Trichomonas vaginalis* and *Neisseria gonorrhoeae*, as well as women with a history of recurrent VVC, were excluded. Furthermore, women with a known history of active autoimmune diseases, positive for Human Immunodeficiency Virus (HIV), Hepatitis B virus (HBV) and Hepatitis C virus (HCV) or other immunocompromised states (such as those undergoing chemotherapy, chronic immunosuppressive drug use, or organ transplant) were ineligible. Other major exclusion criteria included prior use of systemic and/or topical (vaginal or applied to the vulva) antibiotics, antifungals, or anti-*Trichomonas* treatments within 15 days of the baseline visit, as well as recent administration of systemic or vulvovaginal corticosteroids or any other vaginal medication, anticoagulation (e.g., warfarin, heparin) and immunosuppressive drugs, or estrogen replacement therapy. Finally, subjects with poorly controlled diabetes mellitus; chronic diseases such as moderate to severe hepatic or renal disorders, urinary tract infections, a history of cervical or vaginal malignancy, or any other vaginal-vulvar conditions, such as vulvodynia, vestibulitis, vaginismus and radiation-induced vaginitis, that could confound the interpretation of the clinical and microbiological response, were excluded from the study. Because of the observational cross-sectional design, randomization was not applicable. To reduce bias, laboratory investigators remained blinded to clinical status and group allocation until all laboratory testing was completed.

### Collection and processing of vaginal samples

For each participant, two vaginal swabs were collected and soaked in 1 mL of saline. The first swab was used for clinical and microbiological analyses as previously described (Roselletti et al., 2017, 2019b). The presence of *Candida* spp. was initially assessed by microscopic examination, followed by culture of vaginal swabs on CHROMagar *Candida* (VWR International PBI, Milan, Italy). In parallel, the presence of *Streptococcus agalactiae* was evaluated by microscopic examination and culture on blood agar. Species-level identification was performed using MALDI-TOF mass spectrometry (Biomerieux S.A., France). *C. albicans* clinical isolates were stored on beads in glycerol in Microbank vials (Pro-Lab Diagnostics, Cheshire, UK) at −80 °C until use. For each sample, pH was measured using pH-Fix strips (Macherey-Nagel GmbH & Co. KG, Germany). Vaginal wet mount preparations were examined to assess the presence or absence of lactobacilli, polymorphonuclear PMN cells, clue cells, yeasts and pseudohyphae/hyphae. The number of PMN cells per microscopic field was determined and PMN scoring was performed using a semi-quantitative scale ranging from 0 to 2 as previously described: 0, 0 PMN/field; 1, 1–10 PMN/field; 2, 11–40 PMN/field. The second vaginal swab was centrifuged at 10,000×*g* for 15 min. The resulting pellet was resuspended in 200 μL of saline and sent to BMR Genomics (Padua, Italy) for microbiome sequencing and analysis. Following sample processing, ASYMP (*n* = 10) and SYMP (*n* = 12) samples with DNA of sufficient quantity and quality to perform both microbiota profiling and *C. albicans* quantitative PCR analysis were selected for the present study. From the NEG group, a random subset of samples was selected to provide a comparison group of similar size to the combined ASYMP and SYMP cohorts included in the microbiota analysis.

### *In vitro* morphology evaluation

To evaluate the morphology of *C. albicans* clinical isolates *in vitro*, 2 × 10^7^ yeast cells in 500 μL/well in RPMI 1640 were incubated in a 24-multi-well plate at 37 °C for 18 h. Fungal morphology was then microscopically analyzed using an inverted light microscope (Eurotek by Orma, Milan, Italy). Images were captured using a digital camera at 400× magnification.

### Biofilm screening

Biofilm formation was investigated as previously described (Sabbatini et al., 2021). Briefly, single colonies of *C. albicans* isolates cultured on yeast extract peptone dextrose (YEPD) plates were inoculated into 5 mL of YEPD broth, followed by incubation at 30 °C overnight. Cell densities were then assessed by measuring absorbance at OD_600_.

Yeast cultures were diluted with RPMI 1640 (with L-glutamine and 34.5 g/L MOPS, without sodium bicarbonate; pH 7) at 1 × 10^7^ cells/mL, and 200 μL were transferred to each well of a 96-well microtiter plate (Corning Inc., New York, NY, USA). Plates were incubated at 37 °C for 1.5 h under aerobic conditions. The medium was then discarded, and wells were washed twice with 200 μL PBS to remove non-adherent cells. The culture medium was replaced with 200 μL of fresh medium, and plates were incubated again for 24 h under the same conditions.

Biofilm biomass was quantified using the crystal violet staining method as previously described. Absorbance was measured at OD_590_ using a 96-well microplate reader (Tecan, Mannedorf, Switzerland), and biofilm formation capability was determined as reported by Stepanovic et al. Clinical isolates were categorized into weak, moderate or strong biofilm producers based on the calculated OD values for each isolate (Stepanović et al., 2007; Sabbatini et al., 2021).

### Quantification of *C. albicans* load by quantitative PCR

*C. albicans* load was quantified using the *Candida albicans* qPCR Kit (NZYTech Cat. No. MD05851) and Standard (NZYTech Cat. No. MD07021) according to the manufacturer’s recommended protocol. The quantitative DNA standard provided by the kit was serially diluted 10-fold with dilution buffer to generate a standard curve ranging from 2 × 10^6^ to 2 × 10^0^ copies/μL after amplification.

Each qPCR reaction was prepared in a final volume of 20 μL containing 10 μL of 2× NZYSupreme qPCR Probe Master Mix, 2 μL of 10× *C. albicans*/IEC primer/probe mix, 5 μL of DNA template (sample or standard), and 3 μL of nuclease-free water for the no-template control. Reactions were carried out in 96-well optical plates (MicroAmp Optical 96-Well Reaction Plate, Ref. No. 4346907, Applied Biosystems).

Each qPCR run included a positive control to confirm successful amplification, a no-template control to monitor potential contamination, and an internal extraction control to assess DNA extraction integrity and detect potential PCR inhibitors. All *C. albicans* samples and standards were analyzed across three independent experiments. Mean *Ct* values and standard deviations were calculated for each replicate set.

Amplification and fluorescence detection were performed on the QuantStudio™ 3 Real-Time PCR System (Applied Biosystems, Thermo Fisher Scientific, Waltham, MA, USA) using the following thermal cycling conditions: one initial denaturation cycle at 95 °C for 2 min, followed by 40 cycles of denaturation at 95 °C for 5 s and annealing/extension at 60 °C for 30 s.

Fluorescence signals were recorded at the end of each amplification cycle using the FAM channel for detection of the *C. albicans* target gene and the HEX/VIC channel for the internal extraction control. Data acquisition and Ct determination were performed automatically using QuantStudio™ Design and Analysis Software v1.5.1.

A standard curve was generated by plotting *Ct* values against the logarithm of DNA copy number. Sample concentrations were calculated by interpolation from the standard curve. PCR efficiency and correlation coefficient (*R*^2^) values were derived from the curve to assess assay performance.

### 16S rRNA sequencing and bioinformatic analysis

DNA from 43 samples selected for metabarcoding sequencing (NEG, *n* = 21; ASYMP, *n* = 10; SYMP, *n* = 12) was extracted using the Qiagen DNeasy 96 PowerSoil Pro kit on a Qiacube HT instrument. The V3-V4 regions of the 16S rRNA gene were amplified using Illumina-tailed primers (Pro341F: CCTACGGGNBGCASCAG; Pro805R: GACTACNVGGGTATCTAATCC) and sequenced on an Illumina MiSeq platform using Chemistry V3 with a 300PE strategy (Supplementary Table S1).

Bioinformatic analysis was performed using QIIME2 v2023.7. Raw reads were cleaned from primer sequences using q2-cutadapt and then processed with q2-dada2. Reads were trimmed at the 3′ end (forward: 270; reverse: 215), filtered by quality and length, dereplicated, merged and cleaned from chimeras, thus obtaining unique amplicon sequence variants. The resulting feature table was further filtered by ASV length (>370 nt) and by frequency (0.001%) to remove possible sequencing and bioinformatic artifacts (Supplementary Table S2).

Rarefaction analysis was performed on the final feature table to calculate diversity metrics. Alpha-diversity indices included observed features, Shannon index, Pielou’s evenness and Faith’s phylogenetic diversity. Significance among experimental conditions was assessed using the Kruskal–Wallis test. Bray–Curtis, Jaccard and UniFrac distance matrices, both weighted and unweighted, were computed as beta-diversity metrics. PERMANOVA was used to evaluate differences among groups.

Taxonomic assignment was performed using q2-feature-classifier on three pre-trained classifiers derived from 16S sequences collected into GreenGenes v2022.10, SILVA v138 and RDP v19 databases. An experimental taxonomy was created by selecting the best taxonomic classification for each sequence (Qing et al., 2024) This taxonomy was used to determine the community state type of each sample by applying Valencia software to a modified version of Valencia2 centroids (March 2024).

Differential abundance testing was conducted using the ANCOM-BC2 R library (version 2.12.0 on R version 4.5.3) (Peddada and Lin, 2023) cross all taxonomic ranks. The analysis used a taxa prevalence filter of 0.1, did not calculate structural zeros, and applied an FDR significance threshold of 0.1. A fixed-effects model with the formula Status + CST was applied to adjust for clinical status and community state type. Only Status was assessed when the analysis was limited to patients classified as CST III.

Differential abundance results were visualized using barplots generated with ggplot2 (v4.0.2) and combined using the grid, gridExtra (v2.3) and gtable (v0.3.6) R packages. Violin plots depicting data distribution across clinical groups were rendered using pandas (v3.0.1) and seaborn (v0.13.2) Python libraries.

### Statistical data analysis

Vaginal pH and PMN scores were treated as continuous variables, whereas the proportion of women displaying each *C. albicans* morphological form as categorical data. Continuous variables were compared among groups using the Kruskal–Wallis test followed by Dunn’s multiple-comparisons test. Pairwise comparisons for categorical variables were performed using Fisher’s exact test. To account for multiple testing, Bonferroni correction was applied, with statistical significance set at *p* < 0.017.

To assess ecological interactions between microbial species, fungal burden measured by *C. albicans* qPCR, and host clinical parameters including pH, biofilm OD and PMN score, correlation analysis was performed while accounting for the compositional nature of microbiome data.

The raw ASV count table was first aggregated at species level (taxonomy level 7). To mitigate spurious correlations derived from the constant-sum constraint of sequencing data, the raw species-level count matrix was transformed using centered log-ratio transformation after adding a pseudocount of 1 to handle zeros. To focus on biologically relevant taxa and reduce noise, only species with a mean relative abundance greater than 0.1% across the total count table were retained for downstream analysis.

Pairwise associations between CLR-transformed bacterial abundances and clinical variables were evaluated using Spearman’s rank correlation coefficient computed with the Python pingouin (v0.5.5) statistical package; *p*-values were adjusted using the Benjamini–Hochberg procedure to control the false-discovery rate. Correlations were considered statistically significant at FDR-adjusted *p* < 0.05, and correlation matrices were visualized using seaborn (v0.13.2).

To address the sparse and compositional nature of microbiome data, multivariate statistical analyses were performed using the mixOmics R package (version 6.34.0 on R version 4.5.3). Before analysis, the raw count matrix aggregated at species level was filtered to retain robust signals. Taxa with prevalence lower than 10% across all samples were excluded, and only samples associated with the comparison under analysis were retained (NEG vs. ASYMP, NEG vs. SYMP and ASYMP vs. SYMP). A pseudocount of 1 was added to the filtered count matrix, followed by centered log-ratio transformation to project the data into Euclidean space.

Unsupervised principal component analysis was conducted to assess global data structure and identify potential clustering or outliers. Sparse partial least squares discriminant analysis was then employed to identify discriminative microbial signatures between clinical groups.

To prevent overfitting, the sPLS-DA model underwent parameter tuning. The optimal number of components and the number of discriminative features retained per component were determined through grid search using 5-fold cross-validation repeated 1,000 times. Model performance was evaluated by minimizing the balanced error rate using the maximum distance metric. The graphical representation of samples projected onto the sPLS-DA components was generated using the plotIndiv function.

The contribution of selected discriminative taxa was visualized using the plotLoadings function and ranked by mean importance. Hierarchical clustering of selected features and samples was computed and visualized using clustered image maps based on Euclidean distance and complete linkage. Multipanel high-resolution figures were assembled using the grid, gridExtra v2.3 and gtable v0.3.6 R packages.

## Results

### Study population and clinical phenotype

**Figure 1:**
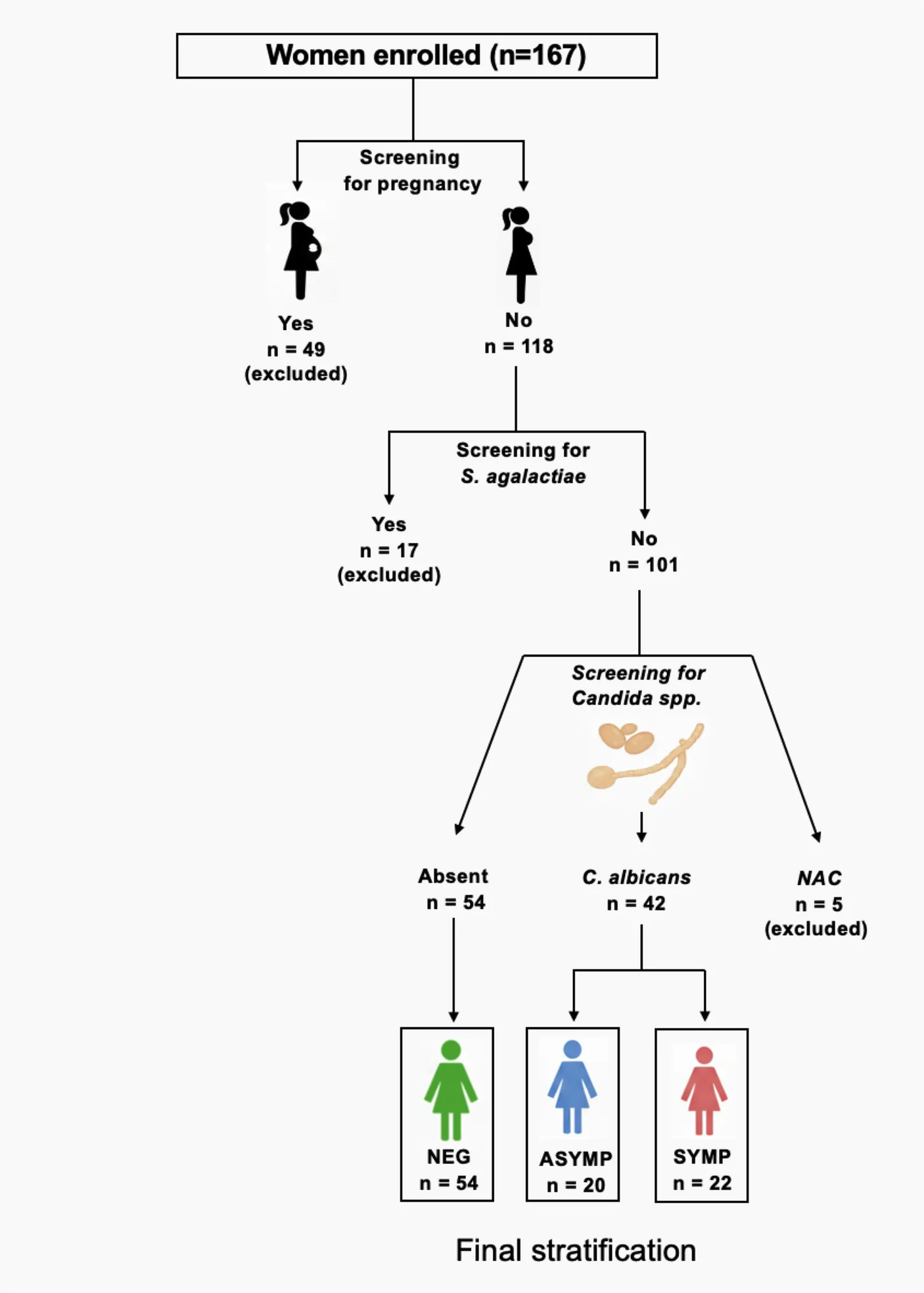
Study population flowchart. Schematic representation of the study enrolment based on pregnancy status, presence of *S. agalactiae*, *Candida* spp., and clinical symptoms. A total of 167 female patients were initially enrolled. Among non-pregnant women, 17 were positive for *S. agalactiae* and 101 were negative. Within the *S. agalactiae*-negative group, 54 showed no presence of *Candida* spp., 42 were positive for *C. albicans*, and 5 were positive for non-*albicans Candida* (NAC) spp. Patients were further categorized according to clinical symptoms into: Negative (NEG, *n* = 54), symptomatic (SYMP, *n* = 22), or asymptomatic (ASYMP, *n* = 20).

Of 167 women screened, 96 were included in the final analytical cohort after exclusion of pregnant participants, women positive for *S. agalactiae*, and women carrying non-*albicans Candida* species. Participants were classified as *Candida*-negative controls (NEG, *n* = 54), asymptomatic *C. albicans* carriers (ASYMP, *n* = 20), or symptomatic VVC patients (SYMP, *n* = 22; Figure 1). The three groups were comparable in age. Clinical signs and symptoms were confined to the SYMP group, which also showed a higher prevalence of vaginal pH ≥ 4.5 and increased PMN scores compared with NEG and ASYMP women (Table 1). *Lactobacillus* spp. were detected by wet mount microscopy in all samples, while clue cells were absent across groups.

**Table 1:** Demographic, clinical, and microbiological characteristics of study participants.

Characteristics	NEG	ASYMP	SYMP
Subject (*n*)	54	20	22
Age (mean ± SD)	32.4 ± 9.15	32.25 ± 9.93	32.13 ± 7.82
Race/ethnicity			
White (non-Hispanic)	47 (87.04%)	19 (95%)	22 (100%)
Hispanic	0	1 (5%)	0
Black	7 (12.96%)	0	0
Asian	0	0	0
Other	0	0	0
Clinical signs and symptoms	No	No	Vaginal discharge, itching, burning sensation, pain, dryness, erythema and dyspareunia
Vaginal pH			
≥4.5	8 (14.81%)	5 (9.09%)	20 (75%)^####^
<4.5	46 (85.19%)	15 (90.91%)	2 (25%)^****^
Wet mount microscopy of vaginal swab			
Clue cells	0	0	0
*Lactobacillus* spp.	54	20	22
*C. albicans* morphology:			
Only hyphae	NA	0	1 (4.5%)
Only pseudohyphae	NA	0	0
Only yeast	NA	13 (65%)	0^####^
Hyphae + yeast	NA	2 (10%)	11 (50%)^#^
Pseudohyphae/hyphae + yeast	NA	5 (25%)	10 (45.5%)
PMN score			
0	39 (72.22%)	14 (70%)	0^****^
1	15 (27.78%)	6 (30%)	1 (4.55%)
2	0	0	21 (95.45%)^####^

NEG, negative; ASYMP, asymptomatic; SYMP, symptomatic; PMN, polymorphonuclear neutrophils; NA, not applicable; *n*, number of samples.

The table summarizes the demographic and microbiological characteristics of the recruited women, grouped according to their *C. albicans* status: negative (NEG, *n* = 54), *C. albicans* positive and symptomatic (SYMP, *n* = 22), and *C. albicans* positive and asymptomatic (ASYMP, *n* = 20). For each group: Age (mean ± SD), race/ethnicity, clinical signs and symptoms, vaginal pH, *C. albicans* morphology, and the presence of *Lactobacillus* spp. are reported. Data are reported as number of samples and percentages or mean ± SD. Statistical significance for vaginal pH and PMN score was determined using the Kruskal–Wallis test with Dunn’s multiple comparisons test. For *C. albicans* morphology, differences were evaluated using Fisher’s exact test. ^****^p < 0.0001 for SYMP vs. NEG (vaginal pH, *C. albicans* morphology, and PMN score); ^####^*p* < 0.0001 for SYMP vs. ASYMP (vaginal pH); ^#^*p* < 0.05 for SYMP vs. ASYMP (*C. albicans* morphology).

*C. albicans* morphology differed between asymptomatic colonization and symptomatic disease. Yeast forms predominated in ASYMP samples, whereas SYMP samples were characterized by frequent hyphal and/or pseudohyphal forms, consistent with the inflammatory phenotype observed in this group (Table 1).

### *C. albicans* morphology and biofilm formation

**Figure 2:**
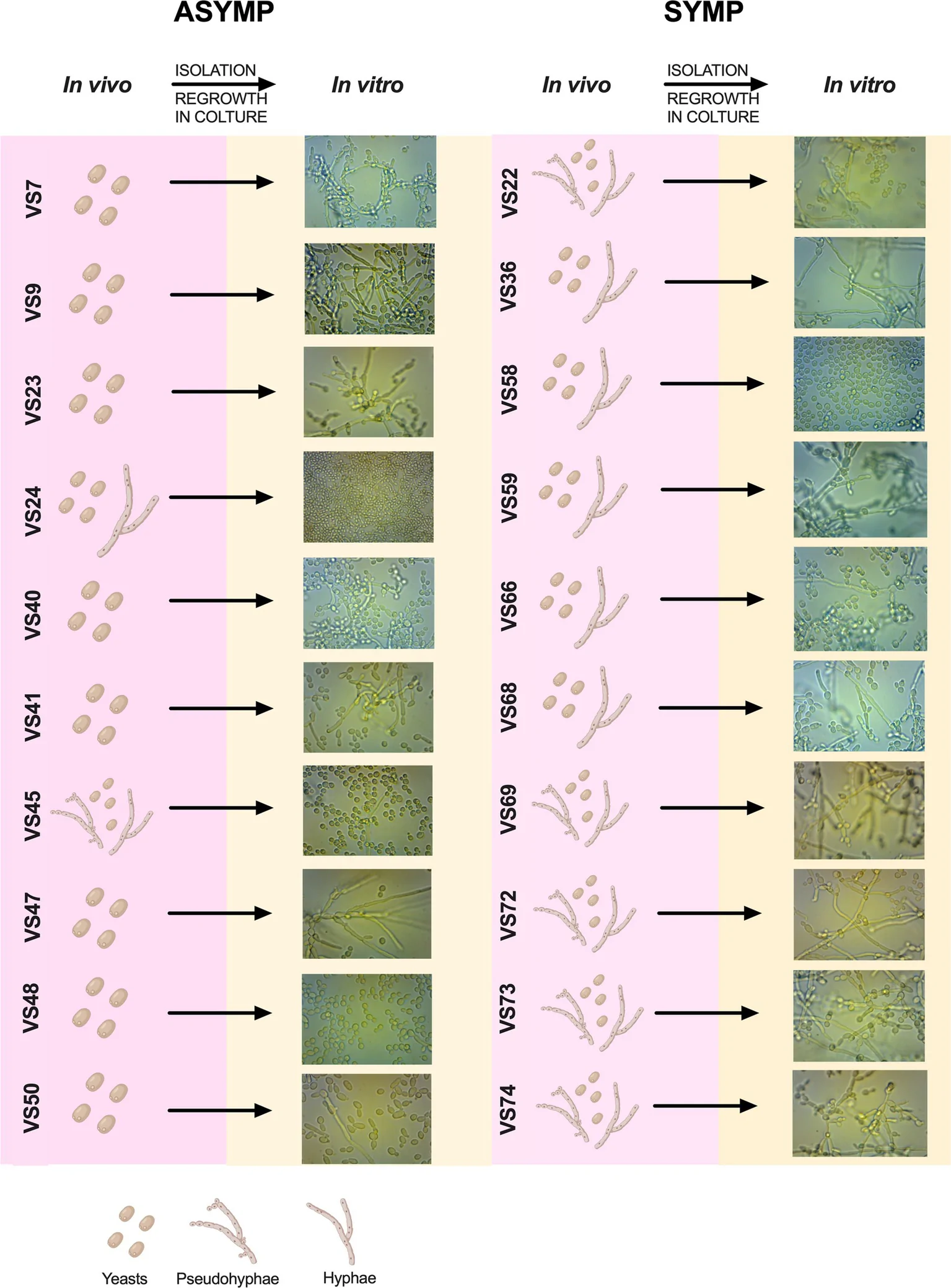
Comparison of *C. albicans* morphology *in vivo* and *in vitro* from ASYMP and SYMP patients. Morphological features of selected *C. albicans* clinical isolates obtained from asymptomatic (ASYMP) and symptomatic (SYMP) women included in the study. For each isolate, the fungal morphology observed *in vivo* (i.e., from the direct observation of the vaginal sample; see also Supplementary Table S3) was compared with the morphology observed after isolation and regrowth in culture (*in vitro*). Representative microscopic images (magnification 200×) from two independent experiments are shown. Illustration partially created with BioRender. Vaginal ASYMP sample size: *n* = 10, SYMP: *n* = 10.

To compare *in vivo* and *in vitro* morphology, ten *C. albicans* isolates from each *C. albicans*-positive group were evaluated after regrowth under standardized culture conditions. Most isolates from both ASYMP and SYMP women formed filamentous structures *in vitro*. However, nine out of ten ASYMP isolates that displayed hyphal morphology *in vitro* were observed predominantly as yeasts in the corresponding vaginal samples, whereas all SYMP isolates displayed filamentous forms *in vivo* (Figure 2; Supplementary Table S3).

**Figure 3:**
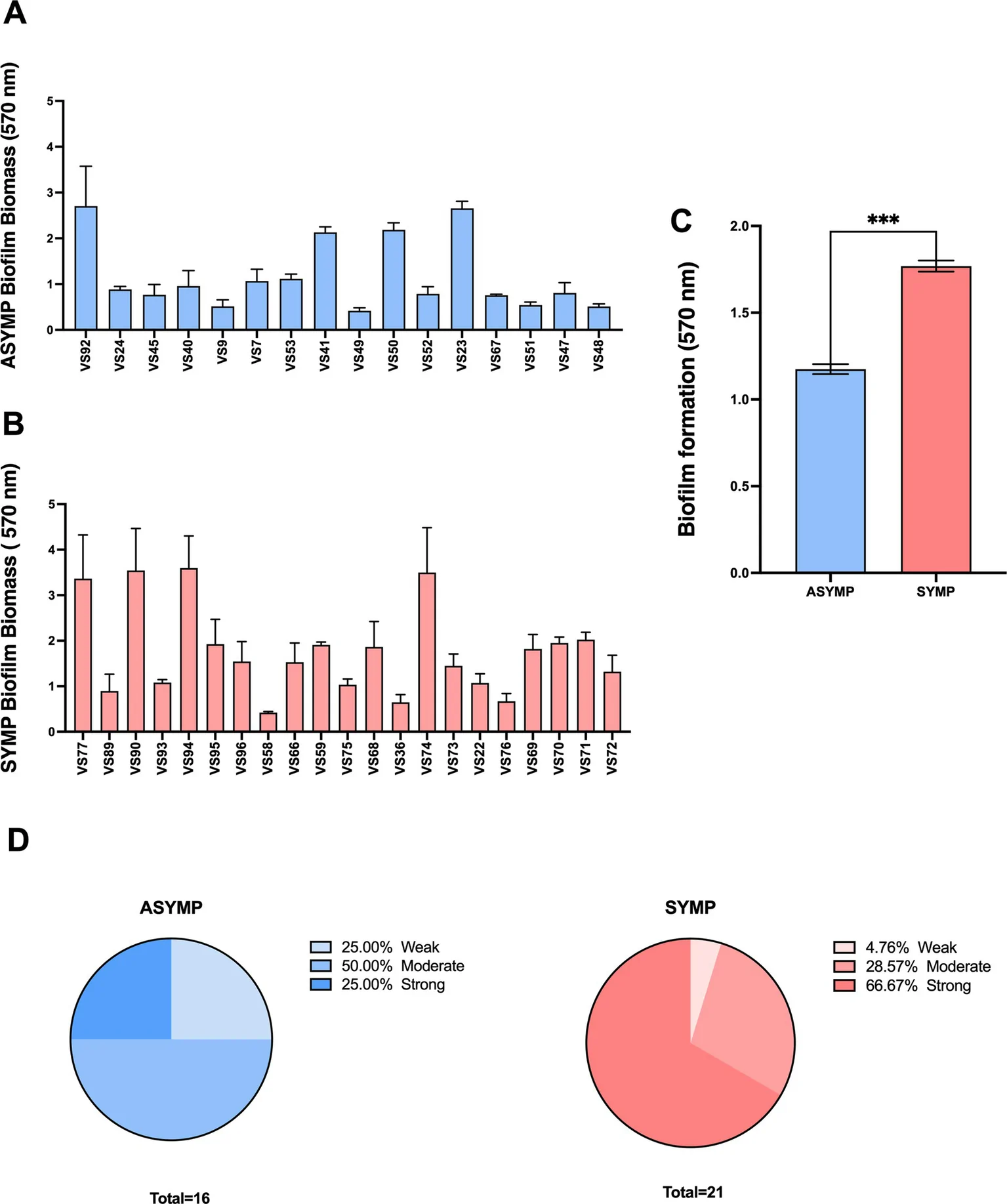
Biofilm production. Biofilm production (measured as *OD*_570 nm_ after crystal violet staining) in selected *C. albicans* clinical isolates from **(A)** ASYMP and **(B)** SYMP women. **(C)** Comparison of mean biofilm formation (± SEM), between ASYMP and SYMP groups. A significant difference was observed between groups (***p < 0.001, Student’s *t*-test). **(D)** Distribution of biofilm-forming capacity among *C. albicans* clinical isolates categorized as weak, moderate, or strong producers in ASYMP and SYMP groups. Vaginal ASYMP sample size: *n* = 16, SYMP: *n* = 21.

Biofilm formation was detected in all recovered *C. albicans* isolates, with substantial inter-isolate variability (Figures 3A,B). Mean biofilm biomass was higher in isolates from SYMP women than in isolates from ASYMP carriers (Figure 3C). Consistently, strong biofilm producers were more frequent among SYMP isolates, whereas ASYMP isolates were mainly moderate biofilm producers (Figure 3D).

### Vaginal microbiota community structure

**Figure 4:**
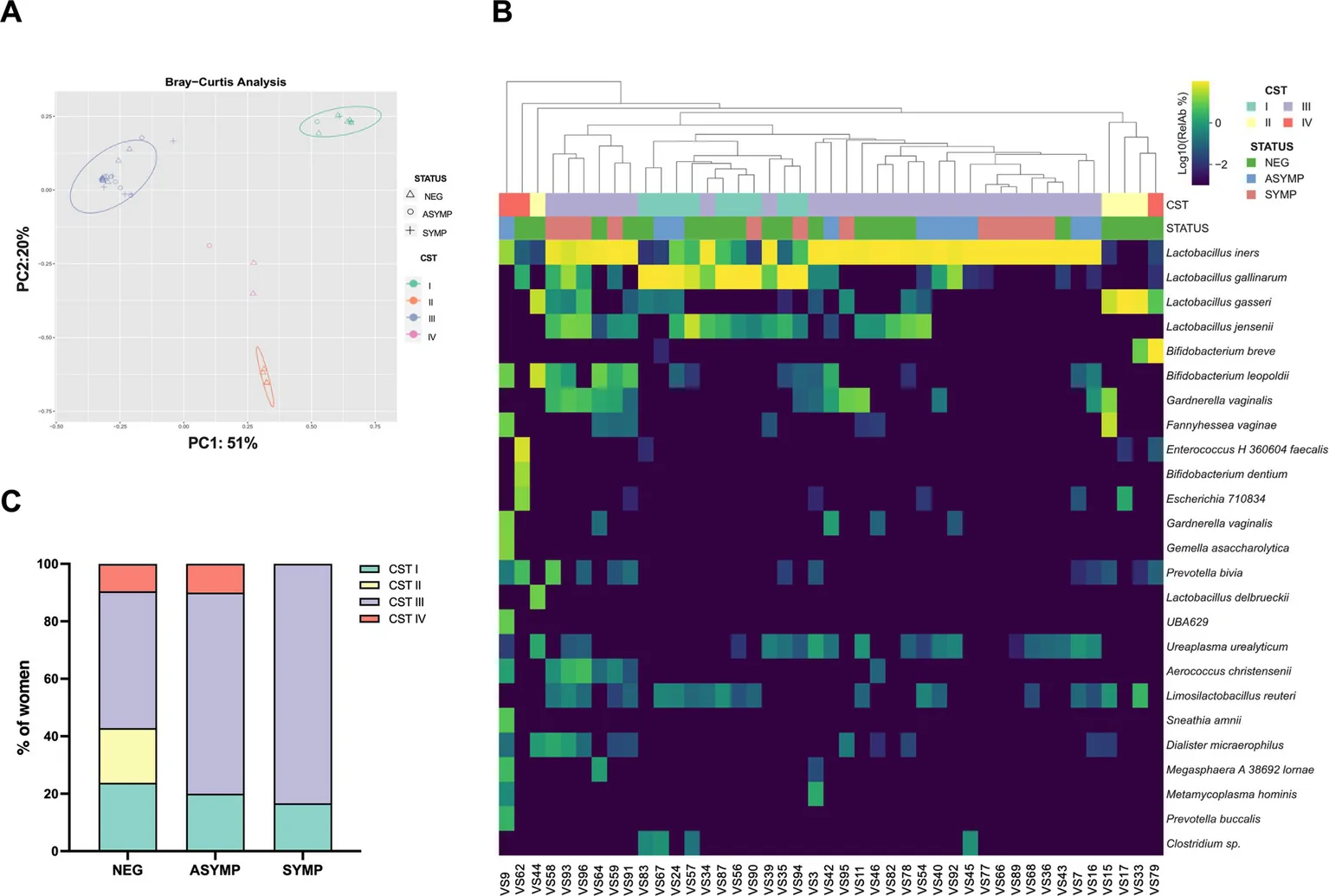
Hierarchical clustering and beta diversity analysis of bacterial species. **(A)** Beta diversity analysis. Principal coordinate analysis (PCA) based on Bray–Curtis dissimilarity matrices. Samples are color-coded by CST using the Set2 palette, while point shapes indicate the clinical status (NEG: Negative; ASYMP: Asymptomatic; SYMP: Symptomatic). Ellipses represent the 95% confidence region assuming a multivariate normal distribution for each CST group. Vaginal NEG sample size: *n* = 21, ASYMP: *n* = 10, SYMP: *n* = 12. **(B)** The heatmap illustrates the log-scaled relative abundance of the 20 most prevalent taxa across all samples. Sample clustering (top dendrogram) was performed using the average linkage method. Color intensity follows the “viridis” scale, ranging from dark purple (low abundance) to yellow (high abundance). Annotation bars above the heatmap indicate the clinical status (color-coded using the Set1 palette) and the assigned community state type (CST; color-coded using the Set2 palette) for each sample. **(C)** Distribution of vaginal community state types (CSTs) in NEG, ASYMP, and SYMP women. Bars indicate the proportion of women assigned to each CST (I–IV) within each study group.

Forty-three vaginal samples were included in the final 16S rRNA gene sequencing dataset (NEG, *n* = 21; ASYMP, *n* = 10; SYMP, *n* = 12). Alpha-diversity indices did not differ significantly by clinical status or CST. In beta-diversity analyses, clinical status did not explain global microbiota clustering, whereas CST assignment was the main driver of community structure. PCA and PERMANOVA indeed showed significant separation among CSTs, particularly for abundance-sensitive metrics, indicating that differences were largely driven by dominant taxa rather than by rare taxa turnover (Figure 4A; Supplementary Table S4). To visualize the bacterial taxa underlying CST stratification, a heatmap based on the most abundant species was generated. Hierarchical clustering highlighted the predominance of *L. iners* in all the three study groups and showed that samples clustered according to their CST classification, consistent with the PERMANOVA results (Figure 4B). In addition, prevalence of CST III increased progressively from NEG to ASYMP and SYMP women (50, 63, and 83%, respectively; Figure 4C; Table 2).

**Table 2:** Pairwise PERMANOVA comparisons between groups.

Group 1	Group 2	Sample size	*F*. Model	*p*. value	*q*. value	Significance
I	II	13	35.627652	0.002	0.003	**
I	III	36	130.911978	0.001	0.002	**
I	IV	12	10.470336	0.005	0.006	**
II	III	31	53.928285	0.001	0.002	**
II	IV	7	3.008567	0.027	0.027	*
III	IV	30	18.578111	0.001	0.002	**

PERMANOVA, permutational multivariate analysis of variance.

Summary of the results of pairwise PERMANOVA comparisons between groups. For each comparison, the sample size, pseudo-*F* statistic (*F*. Model), raw *p*-value, and false discovery rate (FDR) adjusted q-value are reported. Statistical significance is indicated by asterisks: **q < 0.01, *q < 0.05.

### CST III-restricted differential abundance analysis

**Figure 5:**
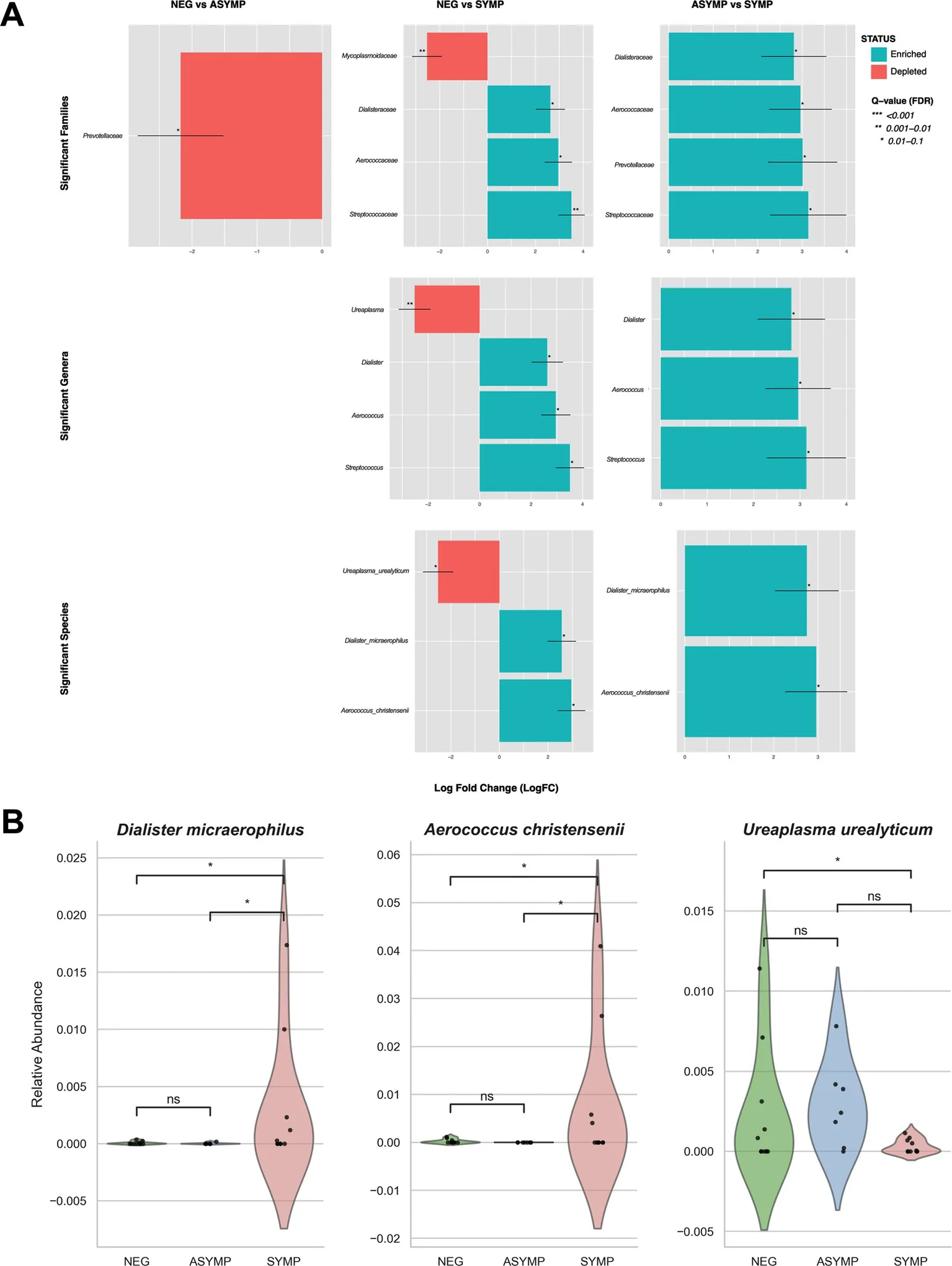
Differential abundance and distribution of key discriminatory taxa across NEG, ASYMP, and SYMP states of women with CST III vaginal microbiota. **(A)** Bar plots representing the differential abundance effect sizes calculated with ANCOM-BC2 across family, genus, and species levels. The *x*-axis displays the log fold change (logFC), while bar fill colours denote the direction of the variation: Cyan indicates enriched taxa, whereas red indicates depleted taxa. **(B)** Violin plots illustrating the data distribution of the relative abundances for each specific taxon. The shape of the violin indicates the probability density of the relative abundance, while fill colours correspond to the women’s clinical status. Asterisks denote the levels of statistical significance based on ANCOM-BC2 adjusted *p*-values (e.g., *q < 0.1, **q < 0.01, ***q < 0.001). Vaginal NEG sample size: *n* = 10, ASYMP: *n* = 7, SYMP: *n* = 10.

To identify microbial signatures specifically associated with clinical status, differential abundance analysis was performed exclusively on CST III samples. *D. micraerophilus* and *A. christensenii* were significantly enriched in SYMP women compared with both NEG and ASYMP groups (Figure 5A), whereas *U. urealyticum* was significantly depleted in SYMP compared with NEG women. In contrast, asymptomatic colonization was not associated with species-level shifts in the vaginal microbiota. Instead, compared with healthy controls, ASYMP women showed a significant depletion of members of the *Prevotellaceae* family. The distribution of relative abundances for all significantly associated taxa across NEG, ASYMP, and SYMP groups is shown in the violin plots (Figure 5B).

### *C. albicans* burden and correlation analysis

**Figure 6:**
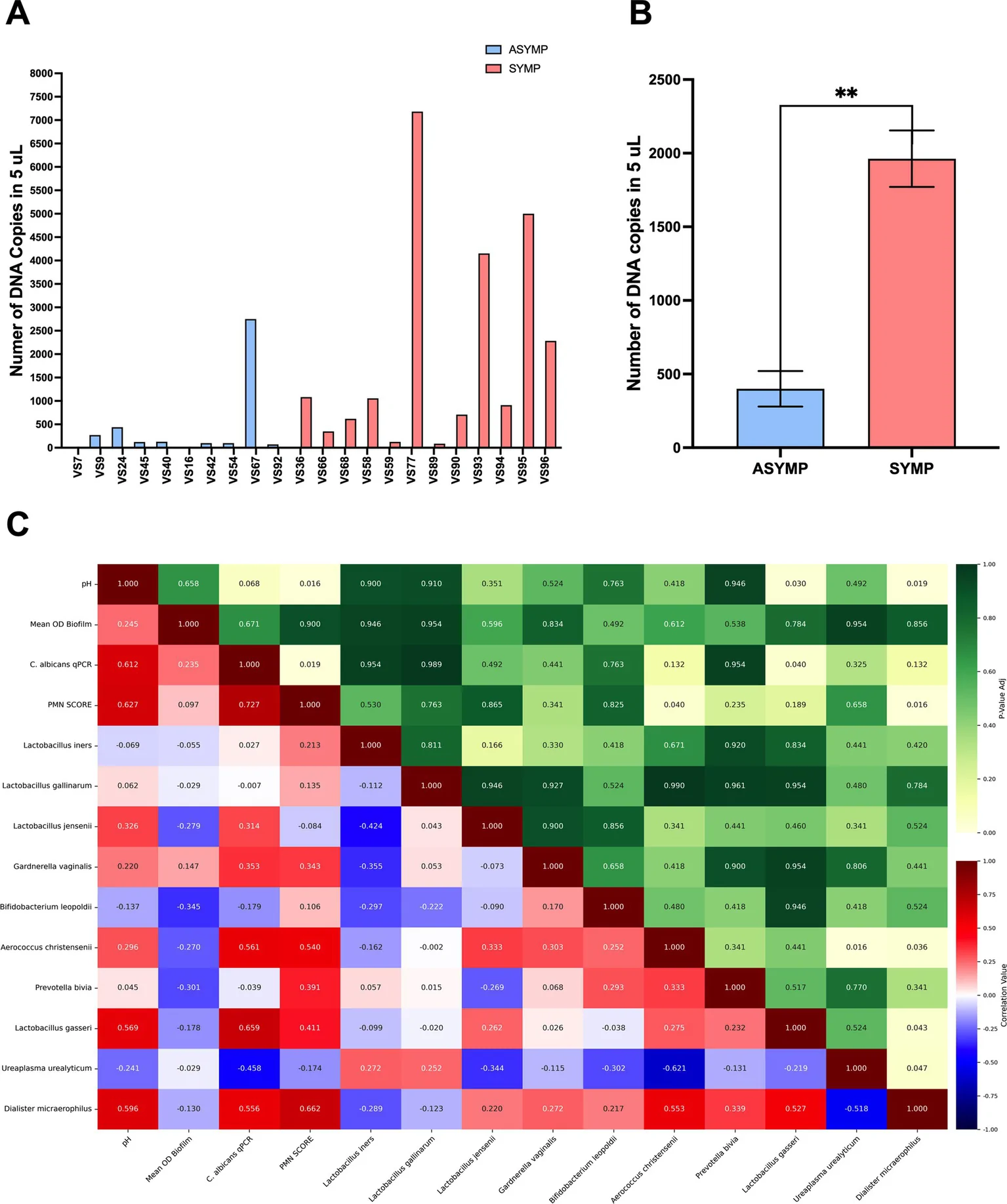
Quantification of *Candida albicans* DNA by real-time qPCR and Spearman correlation analysis. **(A)** Individual *C. albicans* DNA copy numbers (copies per 5 μL) in ASYMP and SYMP samples. Bars represent mean values, and error bars indicate standard deviation (SD) from three independent experiments. **(B)** Comparison of mean *C. albicans* DNA ± SEM between ASYMP and SYMP groups. Vaginal ASYMP sample size: *n* = 10, SYMP: *n* = 12. **(C)** Spearman rank correlation matrix of microbial taxa and clinical metadata within the CST III sub-cohort. Heatmap displaying pairwise Spearman correlation coefficients (*ρ*) between the Centered Log-Ratio (CLR) transformed abundances of the most prevalent bacterial species (mean relative abundance > 0.1%), fungal burden (*C. albicans* qPCR), and host clinical parameters (vaginal pH, PMN score, and mean OD biofilm). The matrix is divided into two sections: the lower triangle visualizes the Spearman correlation coefficients (*ρ*), with colours indicating the strength and direction of the association (Red = Positive, Blue = Negative); the upper triangle reports the corresponding False Discovery Rate (FDR) adjusted *q*-values (Benjamini–Hochberg correction), providing the statistical significance for each pairwise comparison. Vaginal NEG sample size: *n* = 10, ASYMP: *n* = 7, SYMP: *n* = 10.

*C. albicans* burden was quantified in the same samples used for microbiome profiling. SYMP women exhibited a significantly higher fungal load than ASYMP carriers, with a mean *C. albicans* DNA copy number approximately 4.9-fold higher in SYMP samples (*p* < 0.01; Figures 6A,B). To further investigate the relationships between the vaginal microbiota, fungal burden, and host clinical parameters, Spearman rank correlation analysis was performed. *D. micraerophilus* was positively correlated with both PMN score (*ρ* = 0.662, *q* = 0.016) and vaginal pH (*ρ* = 0.596, *q* = 0.019), whereas *A. christensenii* was positively correlated with PMN score (*ρ* = 0.540, *q* = 0.040) but not with vaginal pH (*ρ* = 0.296, *q* = 0.418). In addition, fungal burden was strongly correlated with PMN score (*ρ* = 0.727, *q* = 0.019). A significant positive correlation was also observed between *A. christensenii* and *D. micraerophilus* (*ρ* = 0.553, *q* = 0.036), indicating their frequent co-occurrence. In contrast, *U. urealyticum* showed a distinct pattern, being negatively correlated with both *D. micraerophilus* (*ρ* = −0.518, *q* = 0.047) and *A. christensenii* (*ρ* = −0.621, *q* = 0.016), and with a slight negative correlation, although not significant, with *C. albicans* load (*ρ* = −0.458, *q* = 0.325) (Figure 6C).

**Figure 7:**
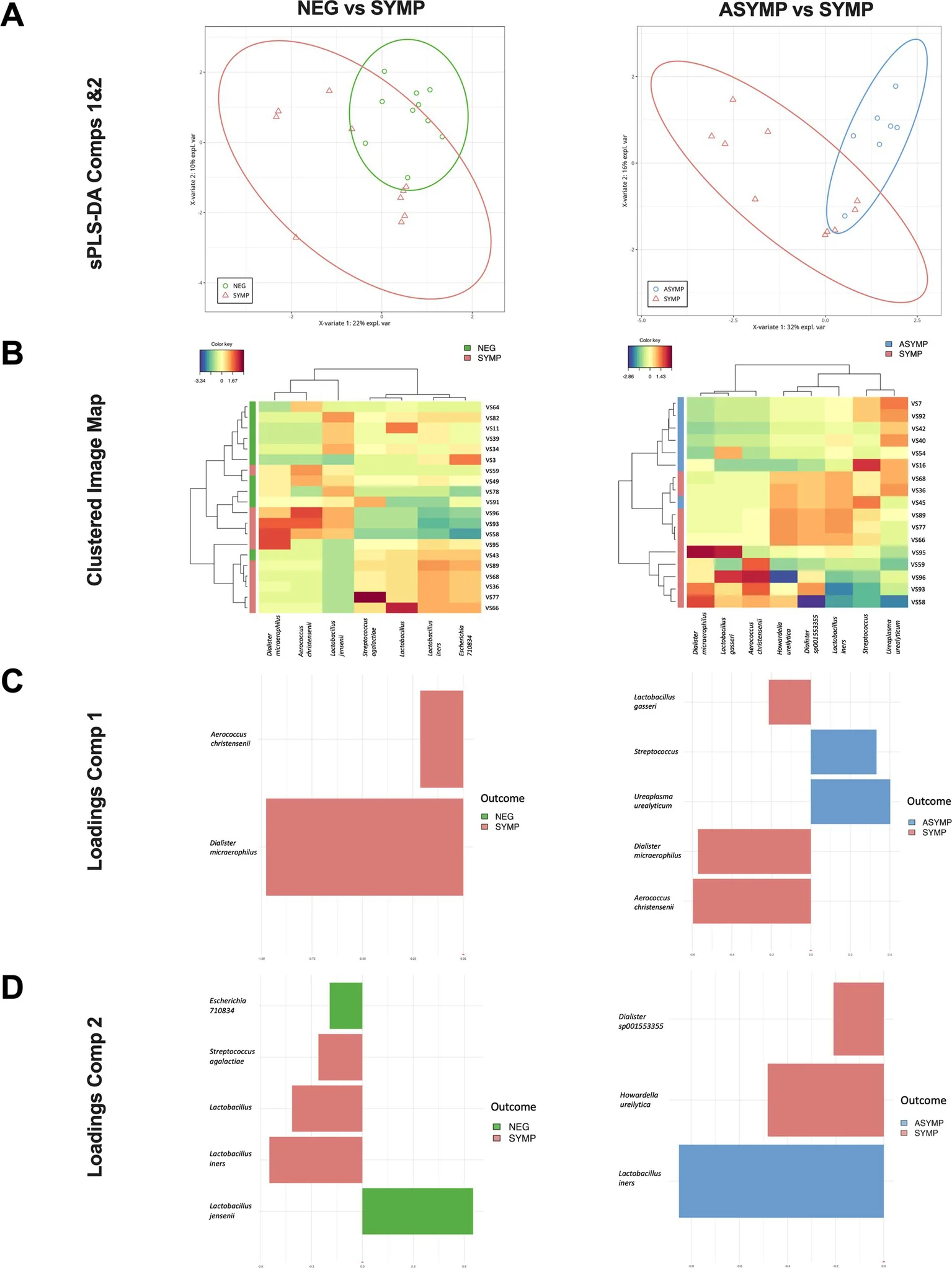
Multivariate and compositional analysis identifying discriminatory microbial signatures between clinical groups. The figure presents the sparse partial least squares discriminant analysis (sPLS-DA) across different pairwise comparisons (e.g., NEG vs. SYMP, ASYMP vs. SYMP). **(A)** sPLS-DA score plots: Sample projections onto the first two latent components. Each point represents an individual sample, with colours denoting the clinical status. Confidence ellipses (95%) highlight structural separation between groups. **(B)** Clustered image maps (CIM): Hierarchical clustering of selected discriminative taxa and samples based on Euclidean distance and complete linkage. The color gradient reflects the CLR-transformed abundances, ranging from relative depletion (blue/dark) to enrichment (red/warm). **(C, D)** Loading weight barplots: Features selected by the sPLS-DA model on component 1 **(C)** and component 2 **(D)**. Bar length represents the discriminative weight (importance) of the taxon for that specific component. Bar fill colours indicate the clinical group in which the taxon exhibits the highest mean abundance, defining its specific association with the respective clinical state. Vaginal NEG sample size: *n* = 10, ASYMP: *n* = 7, SYMP: *n* = 10.

sPLS-DA confirmed that NEG and ASYMP samples had overlapping microbial profiles, whereas SYMP samples separated from both NEG and ASYMP groups (Figures 7A–D). *D. micraerophilus* and *A. christensenii* were the principal taxa characterizing SYMP women, especially in comparisons involving symptomatic disease. The corresponding clustered image maps suggested a distinct symptomatic microbial configuration, consistent with the differential abundance and correlation analyses.

## Discussion

*Candida albicans* is both the main cause of VVC and a frequent commensal of the vaginal niche, indicating that fungal detection alone is insufficient to explain clinical disease (Zangl et al., 2020; Ardizzoni et al., 2021; Gaziano et al., 2023). In this study, the integration of clinical, mycological and microbiome data enabled the identification of specific features associated with symptomatic infection. Symptomatic women exhibited increased vaginal pH, marked neutrophilic infiltration, a higher fungal burden and a predominance of filamentous *C. albicans* forms, whereas asymptomatic carriers resemble *Candida*-negative controls, displaying physiological pH, low PMN scores and predominantly yeast morphology. These findings support the view that symptomatic VVC reflects a breakdown of host-microbe tolerance rather than the mere presence of *C. albicans* (Roselletti et al., 2017, 2019a, 2019b; Kenno et al., 2025).

The *in vitro* analyses further suggest that this pathogenic expression is context dependent. Many isolates from asymptomatic women retain the ability to form filaments under permissive culture conditions, despite being observed mainly as yeasts *in vivo*. Conversely, isolates from symptomatic women are associated with filamentous morphology also in vaginal samples and produce higher biofilm biomass (McKloud et al., 2021; Sabbatini et al., 2021). Thus, *C. albicans* virulence traits appear to be modulated by the vaginal environment, including host inflammatory status and microbial community structure.

Unlike bacterial vaginosis, VVC was not associated with a major loss of *Lactobacillus* dominance (Eastment et al., 2021; Gaziano et al., 2023) or an increase in alpha diversity. CST III, dominated by *L. iners*, emerged as the most prevalent community state type across groups becoming progressively more frequent from *Candida*-negative controls to symptomatic women. This supports a model in which *L. iners*-dominated communities may be permissive for *Candida* colonization, while additional bacterial members could determine whether colonization remains tolerated or progresses to symptomatic inflammation (Ravel et al., 2011; Tortelli et al., 2020; Zheng et al., 2021). A limitation of the CST analysis is the possible misclassification of CST I taxa because V3-V4 16S rRNA sequencing cannot reliably distinguish *L. crispatus* from *L. gallinarum* (Zhang et al., 2019); nevertheless, since *L. gallinarum* is an avian-associated species, rarely found in the human vaginal niche, these sequences can be attributed to *L. crispatus*, confirming a consistent CST association. However, this aspect does not affect the main conclusions, which are based primarily on the well-represented CST III subgroup.

The key finding is the selective co-enrichment of *D. micraerophilus* and *A. christensenii* in symptomatic women, which was associated with host inflammatory markers, particularly PMN infiltration and increased vaginal pH. Specifically, *D. micraerophilus* showed the strongest correlation with inflammation (Sabo et al., 2020), while *A. christensenii* co-varied with the symptomatic microbial profile (Collins et al., 1999; Carlstein et al., 2016; Mollin et al., 2022; Lin et al., 2025). Notwithstanding these strong associations, which suggest that VVC may represent a polymicrobial inflammatory condition, the actual role of specific anaerobic bacteria in amplifying and/or sustaining the host response to *C. albicans* remains to be confirmed. Further validation in larger, independent cohorts is required to establish the value of these taxa as candidate biomarkers for distinguishing symptomatic from asymptomatic women.

**Figure 8:**
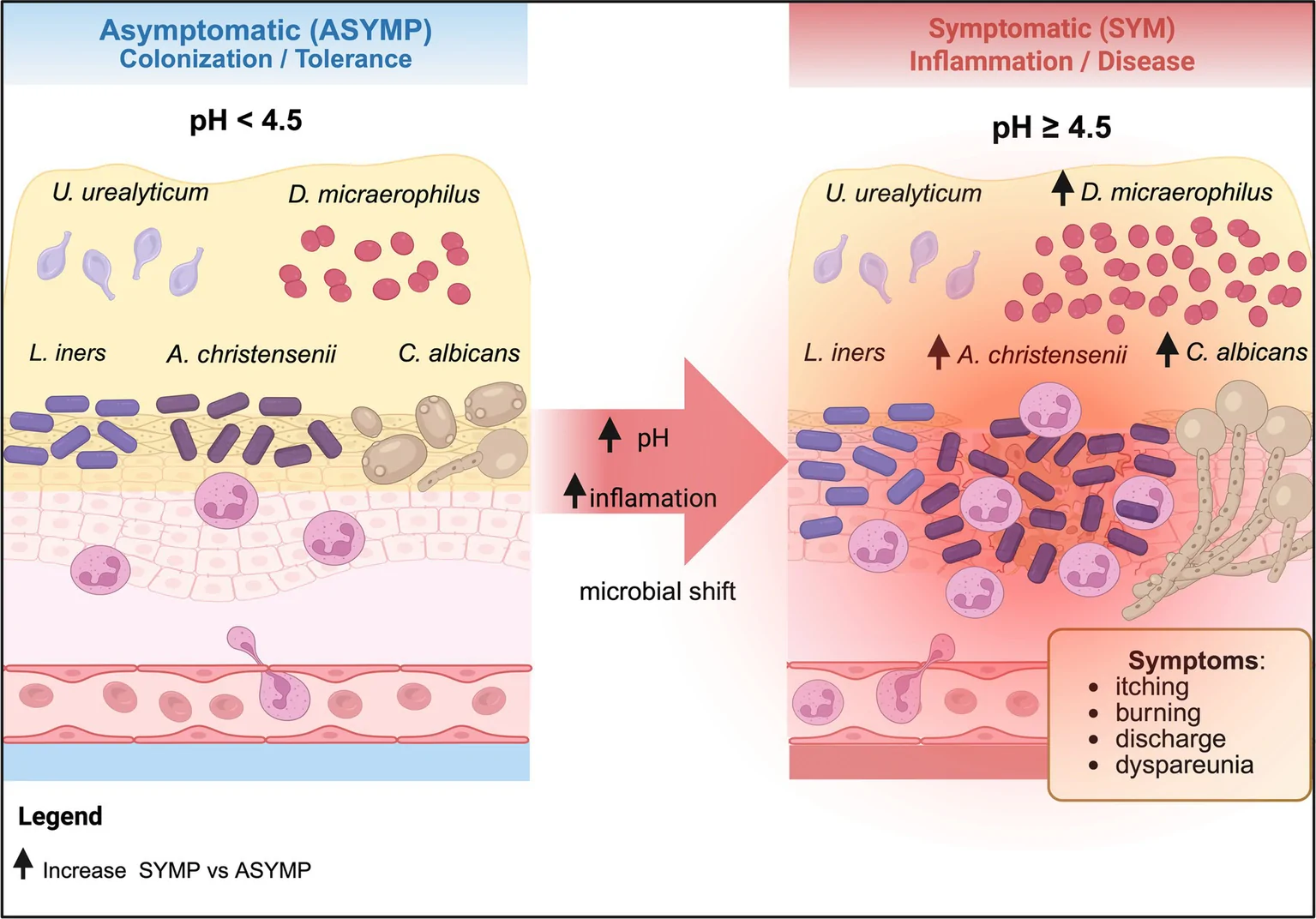
Representation of the co-expansion of *C. albicans* and specific anaerobic bacteria associated with asymptomatic or symptomatic women. Compared with asymptomatic *C. albicans* colonization, symptomatic VVC is associated with a higher abundance of the anaerobic bacteria *D. micraerophilus* and *A. christensenii*, together with fungal filamentation and increased PMN infiltration. Created with BioRender.com.

It is important to note that the results described here derive from a single-centre study with a modest sample size. While sPLS-DA addresses the high-dimensional nature of microbiome data (*p* > > *n*), analysing only 43 women limits statistical power and reduces our ability to detect subtle compositional differences or taxa with small effect sizes, particularly for the ASYMP and SYMP subgroups, where sample numbers are further reduced. An additional limitation is that potential confounding factors that may influence vaginal microbiota composition, including hormonal contraception, menstrual cycle phase, sexual activity, BMI, and probiotic use, were not systematically collected and therefore could not be included as covariates in the analyses. Consequently, we cannot exclude that unmeasured confounding may have influenced the observed associations between bacterial taxa and VVC status. Future studies with larger sample sizes should systematically evaluate these factors to determine their impact on the observed associations. Notwithstanding the acknowledged limitations of the present study (cross-sectional design, lack of external validation, and absence of functional analyses), our findings support the association of *D. micraerophilus* and *A. christensenii* with symptomatic VVC (Figure 8). At present, therefore, these taxa should be regarded as promising candidate biomarkers rather than clinically validated markers. Their clinical implementation will require validation in larger, more diverse independent prospective cohorts representing different ethnicities, geographic locations and socio-economic backgrounds to determine whether they can reliably distinguish symptomatic from asymptomatic women and demonstrate prognostic value, predictive capacity, and added value beyond current diagnostic approaches.

## Data Availability

The datasets presented in this study can be found in online repositories. The names of the repository/repositories and accession number(s) can be found at: NCBI Sequence Read Archive (SRA)/BioProject, accession number PRJNA1499963 (https://www.ncbi.nlm.nih.gov/bioproject/PRJNA1499963).
