# Supplementary material for: Evaluation of Candida albicans and anaerobic bacterial co-expansion in vulvovaginal candidiasis: a cross-sectional study
Source: Front Microbiol. 2026 Sep 3;17:1939688. doi: 10.3389/fmicb.2026.1939688 (PMC13582590; doi:10.3389/fmicb.2026.1939688)
Supplement: Supplementary file 1 [file Table_1.DOCX]

**Supplementary Tables**

# Supplementary Table S1. Primer sequences and sequencing parameters

| **Item** | **Sequence / parameter** | **Notes** |
| --- | --- | --- |
| Pro341F | CCTACGGGNBGCASCAG | Illumina-tailed primer |
| Pro805R | GACTACNVGGGTATCTAATCC | Illumina-tailed primer |
| Target region | 16S rRNA V3-V4 | Metabarcoding sequencing |
| Platform | Illumina MiSeq | Chemistry V3, 300PE strategy |

# Supplementary Table S2. Software, packages, databases and thresholds

| **Category** | **Tool / database** | **Version / threshold** | **Purpose** |
| --- | --- | --- | --- |
| Bioinformatics | QIIME2 | v2023.7 | Main microbiome workflow |
| Primer removal | q2-cutadapt | QIIME2 plugin | Primer sequence removal |
| Denoising | q2-dada2 | QIIME2 plugin | ASV inference and chimera removal |
| Read trimming | Forward 270; reverse 215 | 3' end trimming | Read quality processing |
| ASV filtering | >370 nt; frequency 0.001% | Thresholds | Artifact removal |
| Taxonomy | GreenGenes / SILVA / RDP | v2022.10 / v138 / v19 | Taxonomic assignment |
| CST assignment | Valencia | Modified Valencia2 centroids, March 2024 | Community state typing |
| Differential abundance | ANCOM-BC2 | R library v2.12.0; FDR 0.1 | Differential abundance testing |
| Correlation | pingouin | v0.5.5 | Spearman correlation |
| Visualization | ggplot2 / gridExtra / gtable / pandas / seaborn | v4.0.2 / v2.3 / v0.3.6 / v3.0.1 / v0.13.2 | Figures and plots |
| Multivariate analysis | mixOmics | v6.34.0 | PCA and sPLS-DA |

**Supplementary Table S3: Raw data showing the comparison of *C. albicans* morphology in vivo and in vitro from ASYMP and SYMP patients.** Data represent morphological assessments of individual vaginal samples

|  |  | ***In vivo* morphology** | | | ***In vitro* morphology** | | |
| --- | --- | --- | --- | --- | --- | --- | --- |
| Vaginal samples | Sample Categorization | Yeasts | Hyphae | Pseudohyphae | Yeasts | Hyphae | Pseudohyphae |
| VS7 | ASYMP | X | NA | NA | X | X | X |
| VS9 | ASYMP | X | NA | NA | X | X | X |
| VS23 | ASYMP | X | NA | NA | X | NA | X |
| VS24 | ASYMP | X | X | NA | X | NA | NA |
| VS40 | ASYMP | X | NA | NA | X | X | X |
| VS41 | ASYMP | X | NA | NA | X | NA | X |
| VS45 | ASYMP | X | X | X | X | X | X |
| VS47 | ASYMP | X | NA | NA | X | X | X |
| VS48 | ASYMP | X | NA | NA | X | X | X |
| VS50 | ASYMP | X | NA | NA | X | X | X |
| VS22 | SYMP | X | X | X | X | X | NA |
| VS36 | SYMP | X | X | NA | NA | X | NA |
| VS58 | SYMP | X | X | NA | X | NA | NA |
| VS59 | SYMP | X | X | NA | X | X | NA |
| VS66 | SYMP | X | X | NA | X | X | NA |
| VS68 | SYMP | X | X | NA | X | X | NA |
| VS69 | SYMP | X | X | X | X | X | X |
| VS72 | SYMP | X | X | X | X | X | X |
| VS73 | SYMP | X | X | X | NA | X | X |
| VS74 | SYMP | X | X | X | NA | X | X |

Supplementary Table S4: Distribution of vaginal Community State Types (CSTs) across study groups. The table displays the absolute frequency of samples belonging to the NEG, ASYMP, and SYMP groups, stratified by the four identified CSTs (I, II, III, and IV). The sample size for each clinical group and the overall prevalence of each CST within the cohort, is shown.

| **Status** | **CST I** | **CST II** | **CST III** | **CST IV** | **Total** |
| --- | --- | --- | --- | --- | --- |
| NEG (n) | 5 | 4 | 10 | 2 | 21 |
| ASYMP (n) | 2 | 0 | 7 | 1 | 10 |
| SYMP (n) | 2 | 0 | 10 | 0 | 12 |
| Total (n) | 9 | 4 | 27 | 3 | 43 |

CST = community state type; NEG = negative; ASYMP = asymptomatic; SYMP = asymptomatic; n = number of samples
